# A Novel Homozygous Mutation in the Transient Receptor Potential Melastatin 6 Gene: A Case Report

**DOI:** 10.4274/jcrpe.2254

**Published:** 2016-03-01

**Authors:** Ayça Altıncık, Karl Peter Schlingmann, Mahya Sultan Tosun

**Affiliations:** 1 Denizli State Hospital, Clinic of Pediatric Endocrinology, Denizli, Turkey; 2 University Children’s Hospital, Clinic of General Pediatrics, Münster, Germany; 3 Denizli State Hospital, Clinic of Pediatric Gastroenterology, Denizli, Turkey

**Keywords:** hypocalcemia, hypomagnesemia, infantile seizures, transient receptor potential melastatin 6

## Abstract

Hereditary hypomagnesemia with secondary hypocalcemia (HSH) is a rare autosomal recessive disease caused by mutations in the transient receptor potential melastatin 6 (TRPM6) gene. Affected individuals present in early infancy with seizures caused by the severe hypocalcemia and hypomagnesemia. By presenting this case report, we also aimed to highlight the need for molecular genetic analysis in inbred or familial cases with hypomagnesemia. A Turkish inbred girl, now aged six years, had presented to another hospital at age two months with seizures diagnosed to be due to hypomagnesemia. She was on magnesium replacement therapy when she was admitted to our clinic with complaints of chronic diarrhea at age 3.6 years. During her follow-up in our clinic, she showed an age-appropriate physical and neurological development. In molecular genetic analysis, a novel homozygous frame-shift mutation (c.3447delT>p.F1149fs) was identified in the TRPM6 gene. This mutation leads to a truncation of the TRPM6 protein, thereby complete loss of function. We present the clinical follow-up findings of a pediatric HSH case due to a novel mutation in the TRPM6 gene and highlight the need for molecular genetic analysis in inbred or familial cases with hypomagnesemia.

**WHAT IS ALREADY KNOWN ON THIS TOPIC?**

Hereditary hypomagnesemia with seconday hypocalcemia is a rare disease, which present at infancy with hypocalcemic seizures, is caused by transient receptor potential melastatin 6 (TRPM6) gene mutations.

**WHAT THIS STUDY ADDS?**

In this study, we report a novel mutation in the TRPM6 gene.

## INTRODUCTION

Magnesium is a cofactor for a group of enzymes and transporters. It also plays an essential role in the synthesis of nucleic acids and proteins (1). Intestinal absorption of magnesium mainly occurs in the jejunum and ileum. About 30-40% of the dietary magnesium is absorbed primarily by passive transport (1,2). Regulation of serum magnesium is controlled mainly by renal magnesium reabsorption. Approximately 20% of filtered magnesium is reabsorbed in proximal tubule, 60% in the cortical thick ascending limb of Henley’s loop, and 5-10% in the distal convoluted tubules (3,4).

Hypomagnesemia is defined as a serum magnesium level <1.8 mg/dL (<0.74 mmol/L) (2). Shift of magnesium from the extracellular fluid into cells or bone (Refeeding syndrome, Hungry bone syndrome), increased gastrointestinal or renal loss, reduced absorption, and use of a variety of drugs including antibiotics and chemotherapeutics, may cause hypomagnesemia (2).

Clinical manifestations of hypomagnesemia are carpopedal spasm, muscle cramp, muscle weakness, tremor, convulsions, athetoid movements, and cardiac abnormalities including atrial tachycardia, fibrillation, and supraventricular arrhythmia (1,2).

Hereditary hypomagnesemia with secondary hypocalcemia (HSH) is an autosomal recessive disease caused by the transient receptor potential melastatin 6 (TRMP6) gene mutations. It is characterized by severe hypomagnesemia and hypocalcemia which lead to seizures and muscle spasms presenting in the first months of life (5).

The TRMP6 gene, encoding the epithelial Mg^2^+ channel TRPM6, is mapped to chromosome 9q22. TRMP6 messenger ribonucleic acid, which is expressed in intestinal epithelial cells and kidney tubules, has a crucial role for transcellular Mg2+ absorption from the intestine and from the distal convoluted tubules. Existence of a mutant TRMP6 channel leads to impaired intestinal Mg^2^+ reabsorption and enhanced renal loss (5,6,7,8).

Here, we report the clinical characteristics and genetic analysis of a Turkish inbred girl with HSH due to a novel TRMP6 gene mutation.

## CASE REPORT

**Figure 1:**
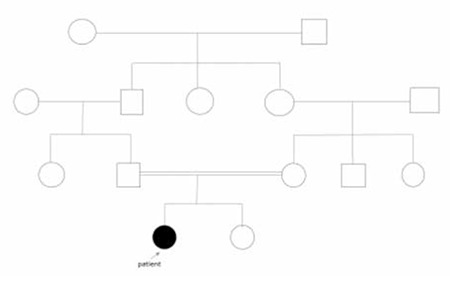
Family pedigree of the patient

The female patient, now aged 6 years, had presented to another clinic with seizures due to hypomagnesemia at the age of 2 months. She was born at term with normal birth weight and length after an uneventful pregnancy. Her parents were cousins. There was no family history of hypomagnesemia, hypocalcemia, or seizures (Figure 1). At the time of her first seizure, total serum calcium was 6 mg/dL (normal, 9-11 mg/dL), potassium 4.1 nmol/L (normal, 3.4-4.5 nmol/L), phosphate 6.3 mg/dL (normal, 2.3-4.7 mg/dL), and magnesium <0.6 mg/dL (normal, 1.6-2.6). Serum vitamin D level was 32 ng/mL (normal, 20-100 ng/mL) and parathormone (PTH) was 5 pg/mL (normal, 15-68 pg/mL). Intravenous Mg2+ sulfate was administered, and she was discharged with a treatment schedule of oral magnesium (elemental magnesium oxide 40 mg/kg/day) and calcium gluconate. Calcium therapy was stopped when the normal calcium levels were achieved. On clinical follow-up, and while receiving oral magnesium therapy, her serum magnesium levels varied between 1.2-1.4 mg/dL, serum calcium levels between 8.5-9 mg/dL, and serum PTH levels between 20-40.2 pg/mL (N=15-68 pg/mL). 24-hour urinary magnesium excretion (Fe Mg2+) was 3.9-5.5% (normal, 3-5%), spot urinary calcium/creatinine ratio was 0.05-0.08 mg/mg (normal, 0.21 mg/mg), and urinary phosphate concentration was 25.1 mg/dL (normal, 40-136 mg/dL). At the age of 3.6 years, the patient has been admitted to our clinic with complaints of chronic diarrhea. She was on magnesium hydroxide therapy, but the daily dose of magnesium varied due to the changes in the severity of her diarrhea. Her weight was 14 kg (-0.94 standard deviation score [SDS]), height was 97.5 cm (-0.69 SDS), body mass index was 14.7 (-0.54 SDS). Midparental height was 156.5 cm (-1.12 SDS). Systemic evaluation was normal and there were no dysmorphic features. Laboratory evaluation revealed a normal complete blood count, as well as normal thyroid, kidney, and liver function tests. Serum total calcium was 8.5 mg/dL, alkaline phosphatase (ALP) 356 U/L, magnesium 1 mg/dL (0.41 mmol/L), and PTH was 43.7 pg/mL (15-68 pg/mL). Spot urinary calcium/creatinine ratio was 0.09 mg/mg (normal, <0.2). Diarrhea was considered to be related with magnesium hydroxide therapy which is normally prescribed as a laxative in constipated individuals, thus the treatment was switched to magnesium oxide tablets at a dosage of 26 mg/kg/day (1 mmoL/kg/day of elemental magnesium) and the stools became normal. On her follow-up for a period of two years, serum calcium levels varied between 8.9-9.2 mg/dL, magnesium levels varied between 1.2-1.4 mmol/dL. Muscle spasms, as reported by the mother, were rare and were thought to be due to the irregular usage of magnesium. A satisfactory growth rate was achieved.

**Figure 2:**
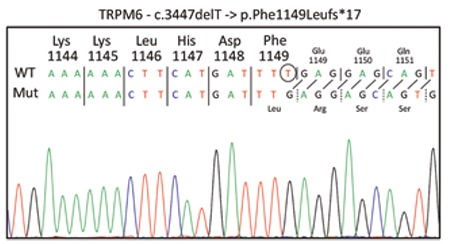
Mutation in the TRMP6 gene

Molecular genetic analysis of TRMP6 was performed by direct sequencing of the coding region and the intron/exon boundaries. A homozygous frame-shift mutation (c.3447delT>p.F1149fs) was identified in the TRMP6 gene (Figure 2). This mutation led to a truncated TRPM6 protein causing a complete loss of function. We have searched the database (http://www.HGMD.cf.ac.uk/ac/index.php) and could not find this mutation. To the best of our knowledge, this is a novel mutation. Genetic counselling and molecular analysis of the parents were planned, but not yet realized.

## DISCUSSION

In this article, we describe the clinical phenotype and follow-up of a patient with HSH due to a novel frame-shift mutation in the TRMP6 gene. Similar to previous reports which state that the onset of the disease is in early infancy at an average age of 4.9 weeks (4-12 weeks), our patient had also presented in early infancy (8,9,10,11,12,13,14,15,16,17,18). Also, similar to previously reported cases, our patient had presented with seizures, a symptom which is the most common manifestation of primary hypomagnesemia in children (8,9,10,11,12). High-dose oral magnesium was successful in our patient to achieve a Mg^2^+ level providing a convulsion-free state. A normal neurodevelopmental outcome and a normal growth were achieved. Failure to thrive and mental retardation are the most frequently reported complications of the HSH. These complications have been attributed to non-compliance to treatment and/or to refractory convulsions due to delayed diagnosis. However, if the patients adhere to the treatment, long-term prognosis seems to be good. Astor et al (19) reported 5 patients without serious complications who had the longest disease duration (over 40 years) in the literature.

Hypomagnesemia in HSH patients results from impaired intestinal magnesium absorption. Active transport of Mg^2^+ via TRPM6 channels situated within the apical membrane of enterocytes prevails when the intestinal Mg^2^+ concentration is low (18). This finding supports that HSH patients fail to absorb Mg^2^+ when the intraluminal Mg^2^+ is low. However, there is a controversy regarding the pathophysiology of urinary magnesium excretion and the role of the magnesium leak. The physiologic range of fractional excretion of magnesium (Fe-Mg^2^+) has been reported to be 3-5% (3). In the presence of hypomagnesemia, Fe-Mg^2^+ is expected to be lower than 0.5-1 % (2,20). Maintaining a Fe-Mg2+ above 2% in the presence of hypomagnesemia is considered as a renal leak (20). In previous studies, initial Fe-Mg^2^+ values of patients with homozygous TRPM6 mutations were reported to be between 0.1-2.3% at the time of diagnosis (9). Additionally, in patients with subnormal serum magnesium levels (1.28 mg/dL), with high dosage of oral magnesium therapy, Fe-Mg^2^+ was reported to vary between 0.2 and 1.6% (8). In contrast to these findings, increased renal Mg^2^+ leak in HSH patients has also been reported (6,10). In our patient, Fe-Mg^2^+ varied between 3.5 % and 5% when serum magnesium levels were subnormal and the patient was receiving magnesium therapy in high doses. This finding supports the role of increased renal Mg2+excretion in the pathophysiology of the disease.

To the best of our knowledge, to date, fewer than 80 cases with TRPM6 gene mutation and 48 different mutations have been reported worldwide (7,8,9,10,11,12,13,14,15,16,17,19). The identified TRPM6 mutations were distributed over the whole gene, without clustering in any specific domain, consistent with the allelic heterogeneity (7,8,9,10,11,12,13,14,15,16,17,19). Genotype-phenotype correlation has not been evaluated properly. Until now, 10 Turkish patients with 7 different mutations were reported. Six of them had splice site and the remaining 4 had non-sense mutations (9,10,11,13). Herein, we report a case who presented with findings consistent with a classical phenotype of HSH and in whom the diagnosis was confirmed by detection of a novel homozygous frame-shift mutation (c.3447delT>p.F1149fs) in the molecular genetic analysis of the TRPM6 gene. Frame-shift mutations have been reported in nine cases with a widespread ethnic distribution including Pakistani, Greek, Indian, and Chinese (10,15). These mutations led to preterm stop codon and loss of function of TRPM6 protein. Reported patients with frame-shift mutations showed similar phenotypic features with a classical clinical presentation of HSH.

In conclusion, HSH is a rare cause of hypomagnesemia. Early diagnosis and proper treatment will prevent complications which may result in irreversible neurological outcomes. Molecular studies in familial or inbred cases with hypomagnesemia are critical to further improve our knowledge of magnesium homeostasis.

## Ethics

Informed Consent: It was taken.

Peer-review: External peer-reviewed.
